# Ephrin-A2 and ephrin-A5 guide contralateral targeting but not topographic mapping of ventral cochlear nucleus axons

**DOI:** 10.1186/s13064-015-0054-6

**Published:** 2015-12-15

**Authors:** Mariam L. Abdul-latif, Jesus A. Ayala Salazar, Sonya Marshak, Minhan L. Dinh, Karina S. Cramer

**Affiliations:** Division of Neonatology, Department of Pediatrics, University of California, Irvine, 101 The City Drive, Orange, CA 92868-3298 USA; Department of Neurobiology and Behavior, University of California, Irvine, 2205 McGaugh Hall, Irvine, CA 92697-4550 USA

**Keywords:** Axon guidance, Medial nucleus of the trapezoid body (MNTB), Calyx of Held, Auditory, Brainstem, Tonotopy

## Abstract

**Background:**

In the auditory brainstem, ventral cochlear nucleus (VCN) axons project to the contralateral, but not ipsilateral, medial nucleus of trapezoid body (MNTB), terminating in the calyx of Held. Dorsal VCN neurons, representing high frequencies, synapse with medial MNTB neurons, while low frequency-coding ventral VCN neurons synapse with lateral MNTB neurons, reflecting tonotopic organization. The mechanisms that ensure strictly contralateral targeting and topographic ordering are incompletely understood. Here we examined the roles of ephrin-A signaling in both types of targeting.

**Results:**

Ephrin-A2 and ephrin-A5 are expressed in VCN cells during late embryonic and early postnatal development. At these ages ephrin-A2 is expressed in axons surrounding MNTB and ephrin-A5 is expressed in MNTB principal neurons. *Ephrin-A2/A5* double knockout mice displayed axon targeting errors in which VCN axons project to MNTB on both sides of the brainstem, where they terminate in calyceal endings. *Ephrin-A2* and e*phrin-A5* single knockout mice showed a similar phenotype. In contrast to effects on contralateral targeting, *ephrin-A2/A5* double knockout mice showed no defects in formation of tonotopically ordered projections from VCN to MNTB.

**Conclusions:**

These findings demonstrate that distinct mechanisms regulate targeting of VCN axons to the contralateral MNTB and targeting to appropriate tonotopic locations. Ephrin-A signaling plays a similar role to ephrin-B signaling in the VCN-MNTB pathway, where both classes normally prevent formation of calyceal projections to ipsilateral MNTB. These classes may rely in part on common signaling pathways.

## Background

Specialized neural circuits in the auditory brainstem underlie the computation of interaural time and intensity differences used in sound localization. In mammals, ventral cochlear nucleus (VCN) neurons receive input from central projections of spiral ganglion cells. VCN globular bushy cell axons cross the midline and terminate in the contralateral medial nucleus of the trapezoid body (MNTB) with large specialized endings known as calyces of Held [1]. MNTB neurons send inhibitory projections to ipsilateral LSO, where the balance of excitation and inhibition is used to determine interaural intensity differences. Central auditory pathways display tonotopic maps reflecting the ordered frequency selectivity of the sensory epithelium [2]. In VCN the dorsoventral axis represents high-to-low best frequencies. Neurons along this axis project topographically to the mediolateral axis of the contralateral MNTB. The VCN-MNTB projection to the appropriate side and tonotopic location reflects specificity that arises initially during axon outgrowth.

During development VCN axons initially reach the midline by embryonic day 13 (E13) and extend to the contralateral MNTB by E17 [3, 4]. The synaptic termination forms at postnatal day 0 (P0) and displays a calyceal morphology at P4-P5 [5–8]. The protracted and orderly growth of these axons reflects the coordinated activity of several families of axon guidance molecules.

Guidance cues include the Eph family proteins, which comprise the Eph receptor tyrosine kinases and their ephrin ligands. These cell surface proteins facilitate cell-cell interactions that play significant roles in axon guidance, cell migration, and other processes [2, 9–11]. This large family of proteins is subdivided into A and B classes based on sequence homology and binding affinity [12]. Ephrin-A ligands bind to EphA receptors and ephrin-B ligands bind to EphB receptors. Crosstalk between the classes emerges from exceptions to this specificity in that ephrin-B ligands bind to EphA4 and ephrin-A5 binds to EphB2 [12–14].

Studies in mutant mouse models have revealed roles for Eph proteins in establishing VCN-MNTB projections and tonotopy. VCN axon projections to MNTB are almost entirely contralateral in the auditory brainstem of wild type mice, but in *ephrin-B2*^*lacZ/+*^ and *EphB2*^*−/−*^*;EphB3*^*−/−*^ mice, which have diminished EphB signaling, a significant number of ipsilateral calyceal projections to MNTB were found [8, 15]. Ipsilateral projections in these mice form at the same time as the normal contralateral projection and do not appear to be eliminated in later maturation. In spite of these significant numbers of aberrant ipsilateral projections, the majority of inputs to MNTB arise as branches from contralaterally projecting VCN axons. Evidence establishing a role for EphB proteins in central tonotopic map formation comes from a study in which mutant mice were exposed to pure tones and patterns of neuronal activation in the auditory brainstem nuclei were examined. Results suggest that ephrin-B2 is needed to form appropriately restricted tonotopic maps in the dorsal cochlear nucleus [16].

Auditory brainstem phenotypes associated with EphB mutations thus show significant effects but suggest that other molecules contribute to specificity in circuit formation. The goal of this study was to evaluate the contributions of EphA signaling. Ephrin-A2 and ephrin-A5 display graded expression levels in retinal axons, and mutations in Ephrin-A2 and ephrin-A5 have been shown to disrupt topographic ordering of projections in the developing visual system [17–20]. In the peripheral auditory system ephrin-A2 and ephrin-A5 are expressed in the cochlea where they regulate afferent axon targeting [21, 22]. Null mutations in *ephrin-A2* and *ephrin-A5* result in frequency-specific abnormalities in auditory brainstem responses that show central as well as peripheral effects [23]. Within the auditory brainstem, ephrin-A5 is expressed in the developing cochlear nucleus and MNTB neurons during embryonic and postnatal ages [8]. Here we examined the function of ephrin-A2 and ephrin-A5 in contralateral target specificity and topographic mapping of VCN projections to MNTB.

## Results

### Developmental expression patterns

#### Ephrin-A2 expression

**Fig. 1:**
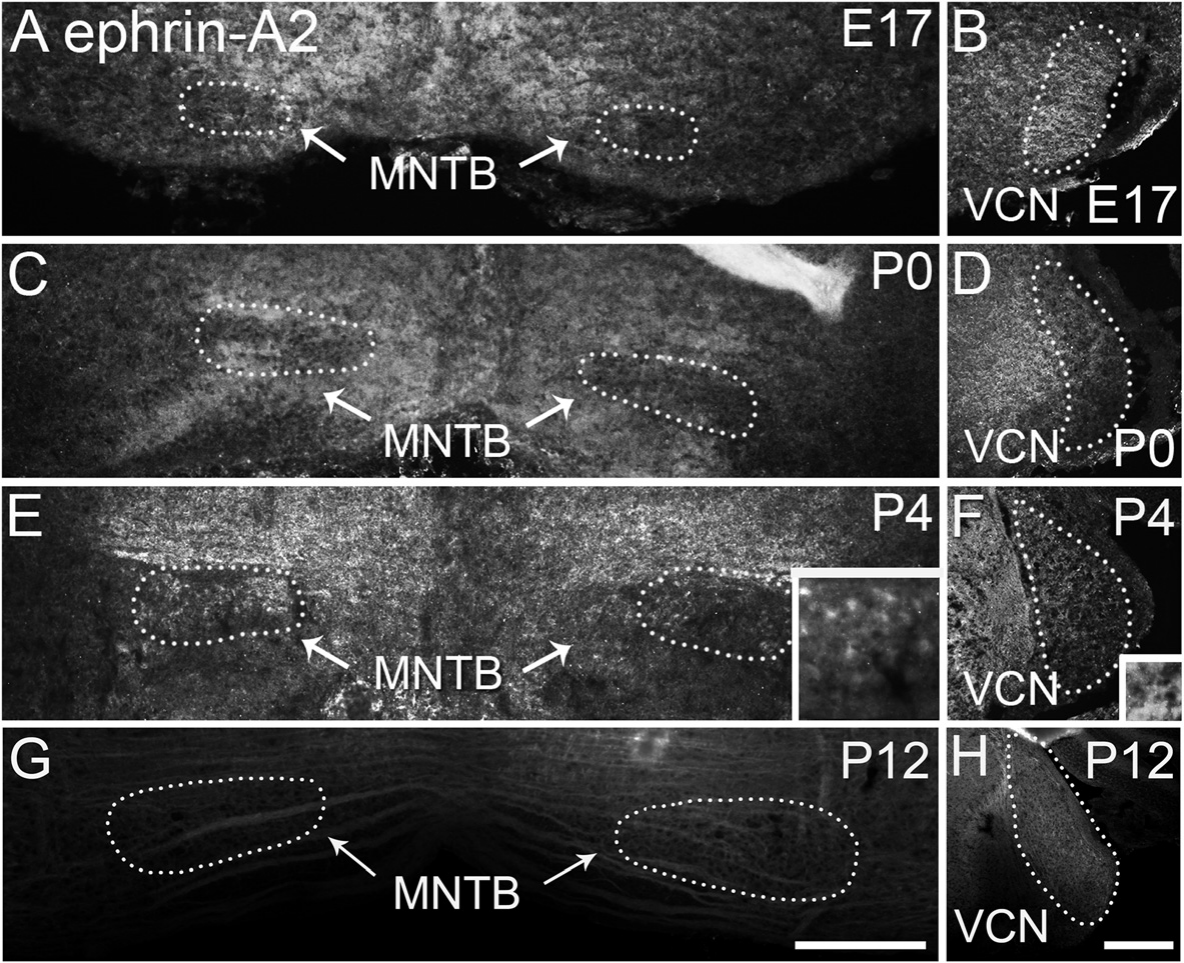
Ephrin-A2 expression in the developing auditory brainstem shown in coronal sections. **a** At E17 ephrin-A2 is expressed in regions surrounding MNTB, with relatively lighter, patchy label within MNTB. **b** At E17 light, fibrous expression of ephrin-A2 is seen throughout VCN. **c** At P0 expression is seen just outside MNTB with sparse expression inside the nucleus. **d** Expression remains in VCN. **e** At P4 expression outside MNTB has increased. Inset shows light label within MNTB. **f** Expression remains in VCN; inset shows fibrous label. **g** Expression of ephrin-A2 is greatly diminished in MNTB at P12. **h** At P12 ephrin-A2 expression is relatively decreased in VCN compared to earlier ages. Scale bar in G, 200 μm, applies to A, C, E, G. Scale bar in H, 200 μm, applies to B, D, F, H. Scale bar in insets, 20 μm

We examined expression of ephrin-A2 in the auditory brainstem during the development of the VCN-MNTB pathway. At E17 ephrin-A2 immunolabeling showed patchy expression in MNTB at low levels, particularly in comparison to the regions surrounding MNTB (Fig. 1a). In VCN ephrin-A2 expression was seen diffusely throughout in a fibrous pattern that did not appear to correlate with cell bodies (Fig. 1b). Similar expression patterns were seen at P0 (Fig. 1c, d). At P4 this pattern continued, with greater expression in the region dorsal to MNTB (Fig. 1e) and similar expression in VCN (Fig. 1f). At P12 very little expression was seen in MNTB (Fig. 1g) and expression in VCN had diminished in comparison to younger ages (Fig. 1h).

#### Ephrin-A5 expression

**Fig. 2:**
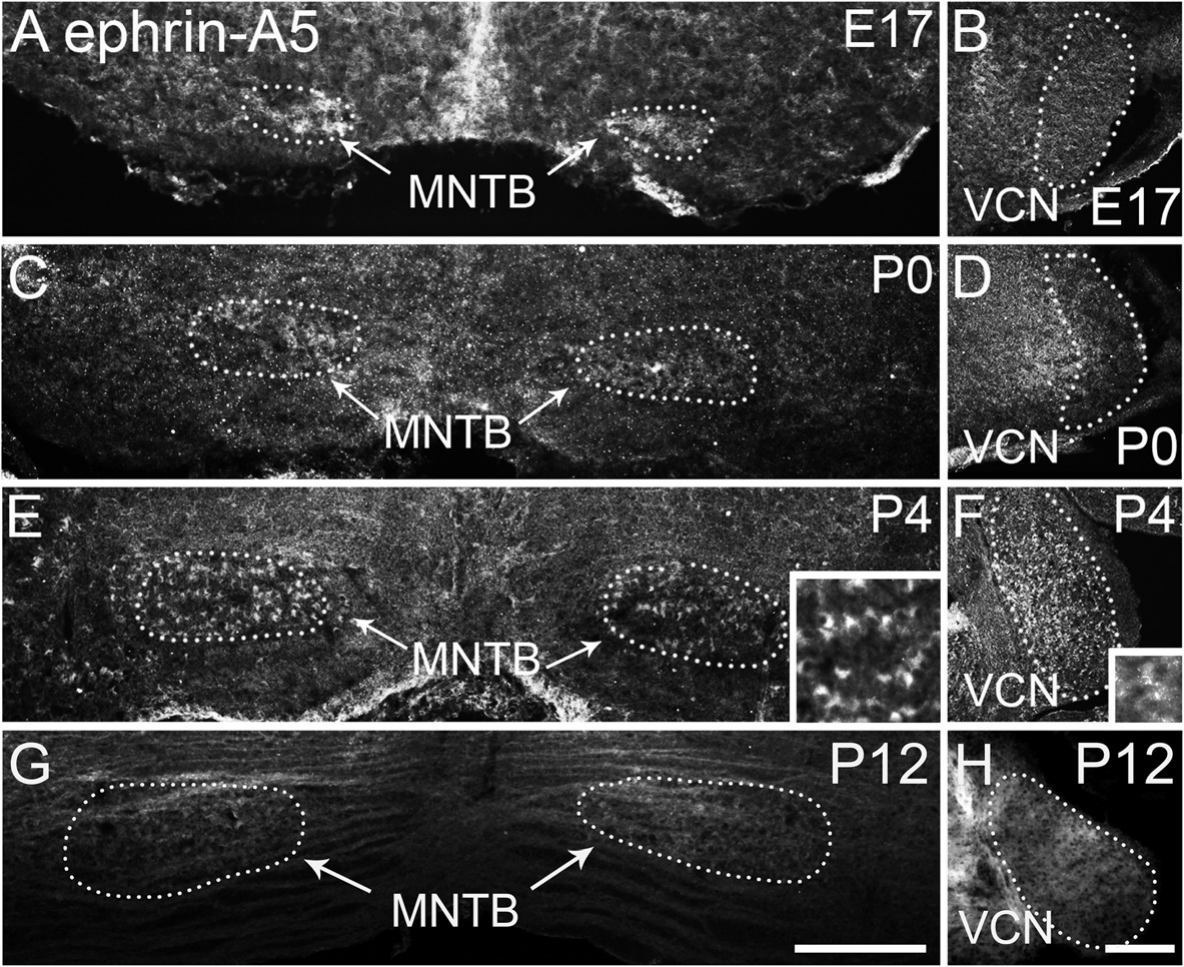
Ephrin-A5 expression in the developing auditory brainstem. **a** At E17 MNTB shows diffuse expression of ephrin-A5. **b** Low expression levels are seen in VCN at E17. **c** At P0 ephrin-A5 is expressed in MNTB. **d** VCN expression has slightly increased at P0. **e** At P4 ephrin-A5 surrounds cell bodies in MNTB (inset) and expression levels have increased. **f** Expression is seen throughout VCN with a diffuse pattern (inset). **g** By P12 immunolabeling shows little expression in MNTB. **h** VCN shows low expression levels at P12. Scale bar in G, 200 μm, applies to A, C, E, G. Scale bar in H, 200 μm, applies to B, D, F, H. Scale bar in insets, 20 μm

Similar to results reported in CD-1/129 mice [8], ephrin-A5 expression was observed in C57BL/6 J mice within MNTB and VCN at E17, P0, and P4 (Fig. 2a-f). At E17 (Fig. 2a-b) and P0 (Fig. 2c-d) expression was sparse and diffuse. At P4, expression appeared most concentrated around cell bodies of MNTB (Fig. 2e). The morphology of labeled regions (Fig. 2e, inset) corresponds with either calyceal terminations on MNTB cells or with MNTB cell surfaces. Expression of ephrin-A5 in both nuclei at P12 was lighter compared to earlier ages.

### *Ephrin-A2* and *ephrin-A5* regulate axon targeting to contralateral MNTB

**Fig. 3:**
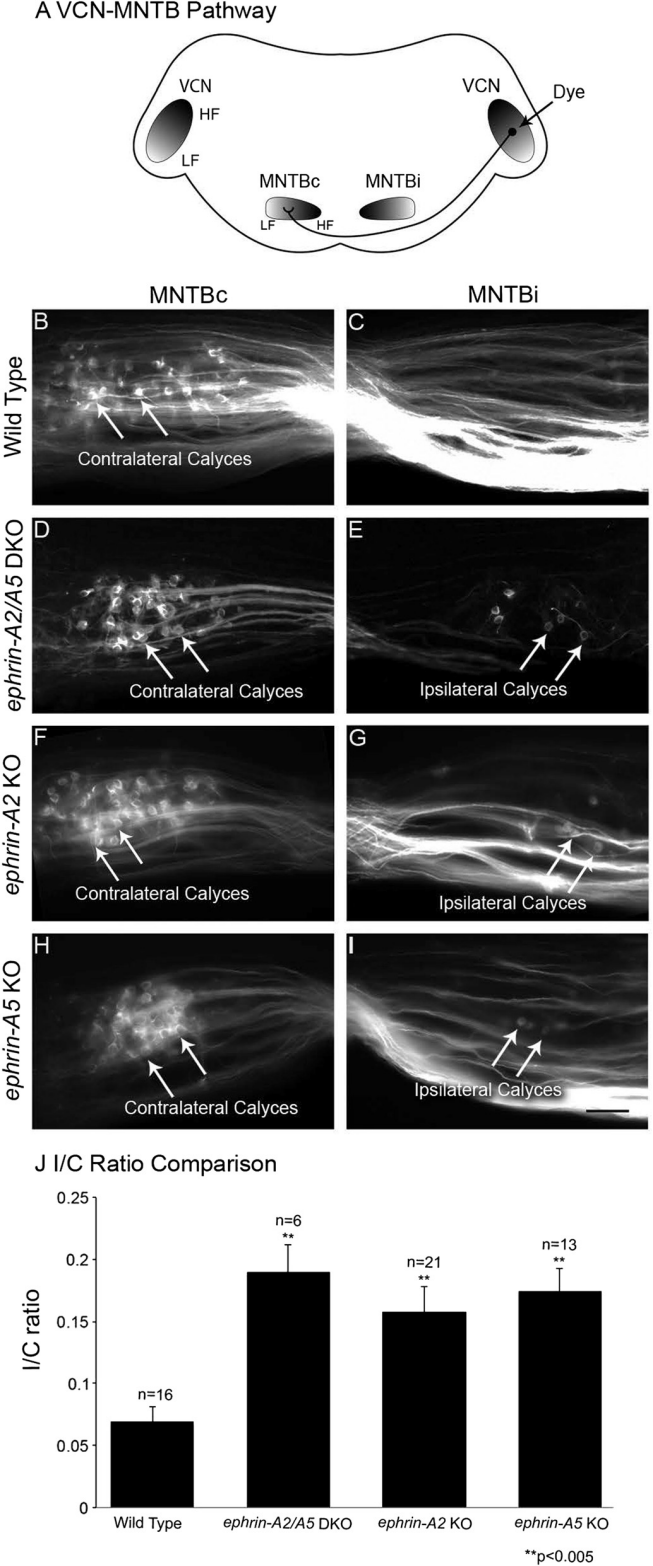
Ephrin-A2 and ephrin-A5 regulate contralateral targeting of VCN axons. **a** Schematic diagram illustrating circuitry in the VCN pathway. Dye placement in VCN on one side normally results in labeled calyces in contralateral MNTB (MNTBc) but not in ipsilateral MNTB (MNTBi). **b** In wild type mice numerous calyces are labeled in MNTBc. **c** Labeled terminations were not formed in MNTBi in wild type mice. **d** In *ephrin-A2/A5* double knockout mice, VCN axons terminated in MNTBc. **e** In addition to the normal projection, *ephrin-A2/A5* double knockout mice also displayed numerous terminations in MNTBi, indicating significant errors in axon targeting. These projections ended in large terminations with morphology similar to the calyx of Held found in contralateral projections. **f, g** *Ephrin-A2* single knockout mice showed a similar phenotype to *ephrin-A2/A5* double knockout mice, with normal contralateral calyces as well as terminations in MNTBi. **h, i** Similarly, *Ephrin-A5* single knockout mice showed both contralateral and ipsilateral calyceal terminations. **j** To compare axon targeting errors between groups we used the ratio of calyces in MNTBi to MNTBc, the I/C ratio. Using ANOVA with *post hoc* analysis, we found a significant increase in I/C ratio for all three mutant mouse groups compared to wild type mice. I/C ratios for mutant mice did not differ significantly from each other. Scale bar in I indicates 100 μm for B-I

To evaluate the role of *ephrin-A2* and *ephrin-A5* in the formation of contralateral axon projections from VCN to MNTB, we performed neuroanatomical tracing in wild type mice and in mice with mutations in *ephrin-A2* and *ephrin-A5.* Lipophilic fluorescent dye was placed in the VCN on one side. After the dye was transported into the VCN axon projections, coronal sections were cut and calyceal terminations could be readily identified in MNTB. Brainstems with adequate label and at least 50 calyces were included in the analysis. The presence of calyces in MNTB both ipsilateral (MNTBi) and contralateral (MNTBc) to the dye placement was evaluated (Fig. 3a). In wild type mice, the majority of axonal terminations from VCN were found in MNTBc and few or no calyces were present in MNTBi (Fig. 3b,c). In brainstem sections of *ephrin-A2*/*ephrin-A5* double knockout (DKO) mice, we observed normal contralateral calyces (Fig. 3d), but in addition we found numerous aberrant ipsilateral projections (Fig. 3e), indicating significant errors in axon targeting. Projections from VCN to MNTBi terminated in a calyceal structure similar to that seen in the normal, contralateral projection. To evaluate the function of each protein individually, mice with single null mutations in *ephrin-A2* and *ephrin-A5* were analyzed. Similar to the *ephrin-A2*/*ephrin-A5* DKO mice, *ephrin-A2* (Fig. 3f, g) and *ephrin-A5* (Fig. 3h, i) knockout (KO) mice both displayed mistargeting of VCN axons with calyces forming in MNTBi as well as in MNTBc.

To quantify the effects of mutations on axon targeting, we counted all the labeled calyces throughout MNTB on both sides of the brain and obtained a ratio of ipsilateral to contralateral calyces (I/C ratio) for each animal (Fig. 3j). For wild type mice (*n* = 16, mean total calyces labeled = 167 ± 56; s.e.m.) the mean I/C ratio was 0.069 ± 0.012, indicating that, as expected, the majority of inputs were seen on the contralateral side. For *ephrin-A2/A5* DKO mice (*n* = 6; mean total calyces labeled = 162 ± 81) the mean I/C ratio was 0.19 ± 0.021. For *ephrin-A2* KO mice (*n* = 21, mean total calyces labeled = 193 ± 97) the I/C ratio was 0.158 ± 0.019, and for *ephrin-A5* KO mice (*n* = 13, mean total calyces labeled = 191 ± 148) the I/C ratio was 0.175 ± 0.018 (Fig. 2j). The total number of calyces measured in each brainstem did not differ among groups (one-way ANOVA; *p* = 0.82). Values for I/C ratios were normally distributed. A one-way ANOVA with Tukey-Kramer *post-hoc* analysis was used to compare the I/C ratios. We found significant differences between the mean I/C ratio of wild type mice compared to *ephrin-A2/A5* DKO mice (*p* < 0.004); *ephrin-A2* KO mice (*p* < 0.002); and *ephrin-A5* KO mice (*p* < 0.001). The I/C ratios did not differ significantly between any of the mutant mouse groups.

### *Ephrin-A2* and *ephrin-A5* mutations do not affect VCN-MNTB topography

**Fig. 4:**
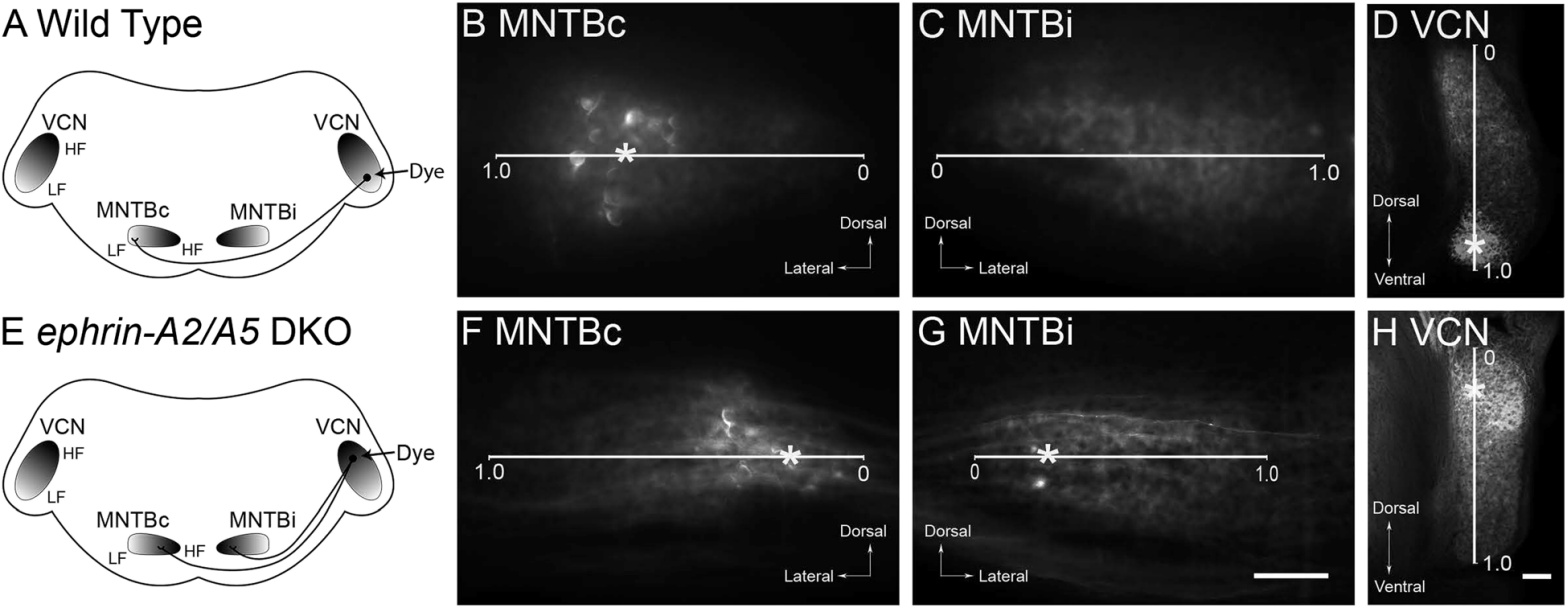
Topographic mapping in wild type and *ephrin-A2/A5* double knockout mice. **a** Schematic diagram showing the expected outcome of ventral dye placements in wild type mice, with labeled calyces seen in the lateral portion of the contralateral MNTB. **b** Labeled calyces in MNTBc after a ventral dye placement in VCN in a wild type animal. The normalized location of a calyx along the mediolateral axis is denoted with an asterisk; normalized positions were averaged to obtain values for each animal. As expected, the calyces are clustered in the lateral portion of MNTBc. **c** No calyces are seen in the MNTBi. **d** Section through the VCN of this animal shows the dye placement in the ventral region, with the center indicated (asterisk). **e** Schematic diagram depicting dye labeling in an *ephrin-A2/A5* double knockout mouse. Dorsal dye placement into the high frequency region of VCN results in labeled terminations in the medial regions of both MNTBc and MNTBi. **f** Labeled calyces in MNTBc after a dorsal dye placement in VCN in an *ephrin-A2/A5* double knockout mouse. Calyces appear clustered in the medial region. **g** Ipsilateral calyces are found in medial MNTB regions after dorsal dye placement in *ephrin-A2/A5* double knockout mice. **h** Section through the VCN of this animal shows the dye placement in the dorsal region, with the center indicated (asterisk). Scale bar in G indicates 100 μm for B, C, F, and G. Scale bar in H indicates 100 μm for D and H

We next tested the role of ephrin-A ligands in the formation of the topographic projection from the dorsoventral VCN axis to the mediolateral MNTB axis, which represent the frequency axes of these nuclei (Fig. 4a). As expected, small deposits of lipophilic dye in the dorsal or ventral VCN of wild type mice (*n* = 16) resulted in labeled calyces in the medial or lateral portion of the contralateral MNTB, respectively. A representative example of a ventral dye placement is shown in Fig. 4b-d.

Focal dye labeling experiments were similarly performed in *ephrin-A2/A5* DKO mice (*n* = 7) and the positions of labeled calyceal terminations in MNTB were evaluated as above. A representative example illustrating dye placement in the dorsal portion of VCN is shown in Fig. 4e-h. Calyceal labeling is seen in the medial portion of both the MNTBc (Fig. 4f) and MNTBi (Fig. 4g).

**Fig. 5:**
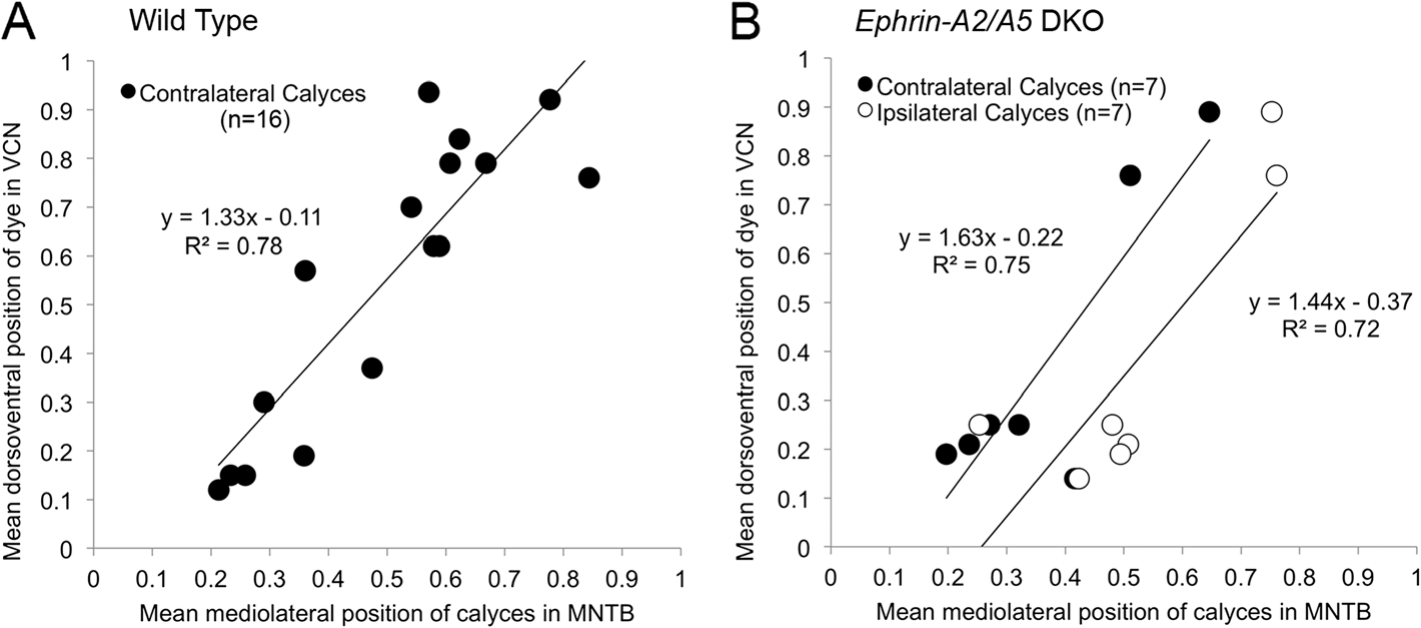
Topographic mapping is similar in wild type and *ephrin-A2/A5* double knockout mice. **a** Scatter plot assessing topography using wild type mice (*n* = 16) shows a strong positive correlation between dye position in VCN and mean normalized position of contralateral calyces, with dorsal VCN regions projecting to medial MNTB, and ventral VCN projecting to lateral MNTB. **b** Analysis of topographic mapping in *ephrin-A2/A5* double knockout mice (*n* = 7). The regression line and correlation coefficient is similar to results seen in wild type mice for projections to MNTBc as well as MNTBi. In these mice the ipsilateral calyces were significantly shifted laterally relative to the contralateral calyces

We used the normalized mean center of the dye placement in VCN across sections to assess the dorsoventral position of the labeled area for each animal. The mean normalized mediolateral position of calyces in MNTB was used as a measure of location of terminations for each animal. We found a strong positive linear correlation between VCN dye position and contralateral MNTB calyx position in wild type animals (regression line y =1. 33x - 0.11; R^2^ = 0.78; Fig. 5a). A scatter plot of the mean dorsoventral VCN dye placement position and resulting mean mediolateral positions of calyces in MNTB showed a strong positive linear correlation for both the ipsilateral (y = 1.63x - 0.22; R^2^ = 0.75) and contralateral (y = 1.43x - 0.37; R^2^ = 0.72) MNTB (Fig. 5b). The slope and correlation coefficients for both MNTBc and MNTBi were similar to results seen in wild type mice, indicating that both ipsilateral and contralateral projections displayed topographic mapping in *ephrin-A2/A5* DKO mice.

We observed a slight lateral shift in the position of ipsilateral calyces compared to contralateral calyces labeled by the same VCN injections. In a two-tailed paired t-test for the comparing mean position of ipsilateral vs. contralateral calyces in *ephrin-A2/A5* DKO mice (*n* = 7), we obtained a p-value of 0.018, indicating that this lateral shift is statistically significant. Together, these observations suggest that *ephrin-A2/A5* DKO mice show normal topographic mapping to contralateral MNTB, and further suggest that aberrant ipsilateral VCN-MNTB projections in these mice follow topographic mapping cues with some differences compared to the contralateral projection.

## Discussion

The projection from VCN to MNTB is almost entirely contralateral, and this precision is disrupted when EphB signaling is inhibited. However, a significant proportion of the projection remains contralateral in mice with loss of EphB signaling [15], indicating that additional mechanisms ensure that VCN axons terminate contralaterally. Here we explored the potential function of EphA signaling. We determined the expression patterns of ephrin-A2 and ephrin-A5 and characterized axon targeting in VCN-MNTB projections of mice lacking one or both proteins. We explored both contralateral targeting and topographic mapping to determine the functions of these proteins.

We found that ephrin-A2 and ephrin-A5 are expressed in the brainstem during embryonic and early postnatal development. Ephrin-A2 is expressed in VCN, where the diffuse label is consistent with neuronal and/or glial labeling. It does not appear to be expressed within MNTB but rather in the fibers surrounding the nucleus. This protein may thus act early on VCN axons and may influence their growth *en route* to their targets in MNTB. Ephrin-A5 is expressed in both VCN and MNTB at P4. In MNTB the labeled region is seen around MNTB cells, a pattern consistent with labeling on the MNTB cell surface or in the calyx of Held. Ephrin-A5 immunolabeling is unlikely to represent glial cells given the fibrous morphology of glial markers in MNTB at early ages [24]. Null mutations in *ephrin-A2* and/or *ephrin-A5* both result in the formation of substantial ipsilateral VCN-MNTB projections in addition to the normal contralateral projection. Despite these similar phenotypes between *ephrin-A2* KO and *ephrin-A5* KO mice, the differing expression patterns suggest that ephrin-A2 and ephrin-A5 proteins act at different points in VCN axon growth. In contrast to these effects on contralateral axon targeting, the *ephrin-A2/A5* DKO mice showed normal topographic mapping from VCN to MNTB on both sides.

### Role of *ephrin-A2* and *ephrin-A5* in targeting to contralateral MNTB

Analysis of VCN projections in mutant mice revealed improper targeting to ipsilateral MNTB when *ephrin-A2* and/or *ephrin-A5* are deleted. This observation is similar to that previously reported for mice with reduced signaling through the ephrin-B signaling pathways. Quantitatively, the I/C ratio values are similar to those of *ephrin-B2*^*lacZ*^ heterozygous mutations and *EphB2/B3* double mutants [15]. This similarity may arise from the two classes of ligands acting on common targets. Ligands are promiscuous within their own class but there is also a degree of crosstalk between classes, and the *EphB2* receptor can bind with *ephrin-A5* ligand [12–14]. Similar phenotypes in mice lacking *ephrin-A5* and *EphB2/B3* suggest that regulation of contralateral targeting relies in part on ephrin-A5-EphB2 binding.

While binding between ephrin-A2 and EphB receptors has not been demonstrated, the similarity in phenotypes in contralateral targeting of the VCN-MNTB pathway suggests that signaling through Eph proteins may converge on a common molecular pathway, possibly through other more complex molecular interactions [2, 25–28]. Additionally, distinct Eph proteins may act at different times, resulting in convergent effects of mutations. Eph proteins influence axon growth and branching at early stages and synaptogenesis at later stages. In the developing MNTB, numerous synapses are formed initially and excess synapses are eliminated during the first postnatal week [29]. It is unclear whether any of the early-eliminated synapses are ipsilateral, but by the time the protocalyx is visible the projection is already nearly entirely contralateral [15]. An interesting possibility is that Eph signaling influences the process of branching in the early MNTB and/or in subsequent synapse elimination. Further evidence for multiple points of regulation lie in the observation that in mutations affecting ephrin-B signaling, ipsilateral projections to MNTB arose either as branches from the contralateral projection of as direct projections from VCN [15]. These effects suggest that some aberrant projections arise from additional proximal branching, whereas others reflect errors in initial axon outgrowth. While the origin of ipsilateral projections is not known for *ephrin-A2* and *ephrin-A5* mutations, the similar phenotype suggests that both trajectories are likely.

### Role of *ephrin-A2* and *ephrin-A5* in topographic targeting

Topographic mapping is seen throughout the central nervous system and represents a fundamental organizing principle of sensory pathways. Topography in a number of pathways has been shown to arise from graded expression of Eph proteins together with varying degrees of activity-dependent refinement [30–32]. In visual system pathways, formation of retinotopy relies extensively on gradients of ephrin-A proteins [33, 34]. In contrast, we found that null mutations in *ephrin-A2* and *ephrin-A5* had no effect on topographic mapping in the VCN-MNTB pathway. Previous studies have suggested that EphB proteins are needed for formation of tonotopy in MNTB [16], the inferior colliculus [32, 35], and the auditory cortex [36]. Our results suggest that ephrin-A2 and ephrin-A5 are not predominant factors in establishment of the VCN-MNTB map in the brainstem. The role of these proteins has not been established in projections from MNTB to its targets, including LSO. The involvement of ephrin-A proteins is not ruled out in other tonotopic projections, as EphA7 modulates tonotopy in the corticothalamic projection from auditory cortex [37]. Moreover, *ephrin-A2/A5* DKO mice show impaired topography in an experimentally induced retinal projection into the medial geniculate nucleus of the thalamus, which normally receives auditory input [38]. These observations suggest a role for EphA signaling in the formation of topography in the auditory thalamus. The roles of other ephrin-A proteins in establishing tonotopy have yet to be identified.

Given that *ephrin-A2/A5* DKO mice have a significant ipsilateral projection, we analyzed this pathway to determine whether the aberrant projection displays topography. We found that topography was similarly strong on the ipsilateral and contralateral sides, indicating that ordering of either projection does not rely critically on ephrin-A2 and ephrin-A5 and instead both rely on other cues. However, a significant lateral shift was noted in the ipsilateral versus contralateral projection in *ephrin-A2/A5* DKO mice. This difference could arise from differences in expression of receptors for graded cues in the target, which could emerge in crossed versus uncrossed portions of VCN axons. Alternatively, the difference could arise in the timing of growth of VCN axon branches to the two sides. Identification of cues that establish tonotopy in this pathway is needed to characterize differences between the normal contralateral projection and the projection on the ipsilateral side.

## Conclusions

Our data show a role for ephrin-A2 and ephrin-A5 in contralateral vs. ipsilateral targeting but not in topographic map formation, indicating that these cues arise from distinct molecules. Conversely, ephrin-B signaling is needed for topographic mapping of projections to the IC, but not for targeting to discrete modular zones within the IC [32]. In retinocollicular pathways, ephrin-A proteins are necessary for topographic map formation, but not for targeting to specific laminae within the superior colliculus [19]. These observations show that Eph signaling can selectively contribute to several distinct dimensions of targeting. The diversity of Eph proteins may thus permit coordinated guidance to correct regions within each target and accommodate multiple rules for connectivity.

## Methods

### Mice

All procedures were approved by the University of California, Irvine Institutional Animal Care and Use Committee (IACUC). We used wild type, *ephrin-A2* KO mice, *ephrin-A5* KO mice and *ephrin-A2/A5* DKO mice, all on strain C57BL/6 J. Double knockouts were obtained by breeding *Efna2*^*tm1Jgf*^*Efna5*^*tm1Ddmo*^/J transgenic mice (Jackson Laboratories), which contained homozygous deletion of *ephrin-A2* and were heterozygous for *ephrin-A5*.

To determine animal genotypes, mice were anesthetized with isoflurane. DNA was then extracted from tail samples as described previously by [15]. Three primers were used for the *ephrin-A2* allele: oIMR8356, 5′-CCG CTT CCT CGT GCT TTA CGG TAT C-3′; oIMR8357, 5′-GGC TAT ACC GTG GAG GTG-3′; and oIMR8358, 5′-CTG CCG GTG GTC ACA GGA-3′. The wild type PCR product is 110 bp while the mutant product is 650 bp as reported by Jackson Laboratory.

The three primers used for the *ephrin-A5* allele: oIMR8359, 5′- ATT CCA GAG GGG TGA CTA CCA CAT T-3′; oIMR8360, 5′- TCC AGC TGT GCA GTT CTC CAA AAC A-3′; and oIMR8361, 5′- AGC CCA GAA AGC GAA GGA GCA AAG C-3′. The wild type PCR product is 397 bp while the mutant product is 513 bp as reported by Jackson Laboratory.

### Immunofluorescence

Wild type mice at ages E17, P0, P4, and P12 mice were used for immunofluorescence. Embryonic tissue was immersion fixed in 4 % paraformaldehyde (PFA) in phosphate-buffered saline (PBS) at 4 °C. P0 and P4 mice were perfused transcardially with 0.9 % saline followed by 4 % PFA in PBS. P0 and P4 brainstems were then dissected and post-fixed for 2 h in 4 % PFA in PBS at 4 °C. Samples were cryoprotected in 30 % sucrose in PBS at 4 °C. Brainstems were sectioned in the coronal plane at 18 μm on a cryostat (Leica Microsystems) and mounted onto chrome-alum-subbed slides. Slides were outlined in PAP pen (Life Technologies) to confine reagents and warmed at 37 °C on a slide warmer for 20 min as described previously [24]. Slides were rinsed in PBS for 30 min and then incubated 1 h in blocking solution consisting of 4 % bovine serum albumin and 0.1 % Triton X-100 in PBS. The primary antibody for ephrin-A2 (5–15 μg/mL in blocking solution) was a goat polyclonal antiserum generated using a recombinant mouse ephrin-A2 protein (aa 1–184, R&D Systems). The primary antibody for ephrin-A5 (5 μg/mL) was a rabbit polyclonal antiserum derived from the C-terminal end of the mouse ephrin-A5 protein (aa 160–250, Invitrogen). Slides were incubated overnight at room temperature with primary antibody, rinsed in PBS for 30 min and then incubated in secondary antibody for 1 h at room temperature. Secondary antibodies used were Alexa Fluor 488 donkey anti-goat for ephrin-A2 and Alexa Fluor 594 goat anti-rabbit for ephrin-A5 (1:300, Life Technologies). Slides were rinsed in PBS for 30 min and coverslipped with Glycergel mounting media (Dako). We confirmed the specificity of the primary antibodies by including control sections derived from *ephrin-A2* KO mice or *ephrin-A5* KO mice for the ephrin-A2 and ephrin-A5 antibodies, respectively; no labeling was seen in these sections.

### Neuroanatomical labeling

Wild type, *ephrin-A2* KO mice, *ephrin-A5* KO mice, and *ephrin-A2/A5* DKO mice were used at P10-P14 for analysis of contralateral targeting. Wild type and *ephrin-A2/A5* DKO mice were used at P10-12 for analysis of topographic mapping. Mice were perfused transcardially with 0.9 % saline then 4 % PFA in PBS. Brainstems and cerebellum were extracted and post-fixed in 4 % PFA in PBS at 4 °C for 24–72 h. The cerebellum was dissected away and a small piece (100–200 μm^2^) of the lipophilic NeuroVue Red dye (Polysciences) was then placed in VCN on one side as described previously [8, 39]. For studies of topography, a smaller piece of NeuroVue Red dye was placed on one side of either dorsal or ventral portion of VCN. Brainstems were then returned to 4 % PFA in PBS and incubated at 37 °C for 2 weeks to allow for dye to transport along the axon and into the calyces on MNTB. Brainstems were placed in 4 % low-melting agarose in PBS after incubation and sectioned coronally at 100 μm on a vibratome (Leica Microsystems). Sectioned tissue was then mounted onto chrome-alum-subbed slides and coverslipped.

### Imaging

Immunofluorescence images of brainstem sections were acquired with a Carl Zeiss Axioskop microscope, Axiocam digital camera and Axiovision software (Zeiss). Images were then imported into Adobe Photoshop CS6 v13.0 for brightness and contrast optimization. The boundaries of VCN and MNTB were visualized in green autofluorescence images.

### Analysis of contralateral targeting

The specificity of the VCN-MNTB pathway was analyzed by examining the number of calyceal terminations from the labeled VCN to both contralateral MNTB and ipsilateral MNTB, where the large calyceal terminations can be clearly visualized and counted. Analysis was performed blind to genotype. Samples were included in the study if (1) labeled axons originated from an intact VCN and coursed along the ventral region of the midline; (2) axons terminated at calyces within MNTB; (3) calyceal terminations covered at least one-fourth of the cell surface in MNTB; and (4) at least 50 total labeled calyces were found in the brainstem. To account for variations in dye labeling across animals, we computed an ipsilateral to contralateral ratio (I/C ratio) as described previously [8, 15, 39, 40] by dividing the total number of MNTBi projections by the total number of MNTBc projections to obtain a single value for each animal. A one-way ANOVA with post-hoc analysis was used to compare the I/C ratios.

### Analysis of topographic mapping

Brainstems were included in the analysis of topographic mapping if (1) dye extended 50 % or less than the total dorsoventral length of VCN; (2) dye was transported along the axon from VCN and terminated in calyces; and (3) dye labeled at least 10 calyces total in MNTBi and MNTBc. Brainstems were excluded if dye was located on parts of brainstem other than VCN or if no calyces were labeled in MNTBc. Analysis was performed blind to genotype.

Single 10x magnification green and red fluorescent images of VCN were taken to measure the ventral to dorsal length of VCN and dye mean center using Axiovision software (Zeiss). VCN was identified under green autofluorescence and the most dorsal point of VCN was set as 0 μm per section. The average length for each brainstem was calculated. The borders of dye placement along the ventral to dorsal axis of VCN were determined using the red fluorescence image and the midpoint of the dye extent was used to determine the dye position relative to the 0 μm reference:

$$ VCN\  dye\  position = \left(\frac{ventral\  dye\  border\ \hbox{-} dorsal\  dye\  border2}{}\right) + dorsal\  dye\  border $$

The VCN dye position was normalized to the dorsoventral length of VCN. This normalized dye position was averaged across all VCN sections containing dye labeling to obtain a single value for each animal.

The average dye extent was calculated to determine inclusion criteria for each brainstem:

$$ Avg\ VCN\  dye\  extent=\frac{mean\  ventral\  border\hbox{-} mean\  dorsal\  border}{average\ VCN\  length}\ X\ 100 $$

Specimens with values 50 % and lower were included. Z-stack 20x green and red fluorescent images were obtained for MNTBi and MNTBc. The length of the ipsilateral and the contralateral MNTB was measured in each section from the most medial position to the most lateral position using the green fluorescent image. The positions of labeled ipsilateral and contralateral calyces from the medial border MNTB were measured using the z-stack red fluorescent images. For each calyx, the distance from the medial border was normalized to the mediolateral width of MNTB in that section. The average normalized calyx position in MNTBi and MNTBc was calculated for each brainstem.

A scatter plot of the VCN normalized dye position and the MNTB normalized calyx position was constructed to assess topographic mapping in wild type and *ephrin-A2/A5* double knockout mice. Linear regressions were used to assess the correlation between the location of the VCN dye placement and locations of calyces in MNTB.

## Abbreviations

MNTBc: Contralateral medial nucleus of the trapezoid body
MNTBi: Ipsilateral medial nucleus of the trapezoid body
I/C: Ipsilateral to contralateral
MNTB: Medial nucleus of the trapezoid body
PFA: Paraformaldehyde
PBS: Phosphate-buffered saline
VCN: Ventral cochlear nucleus
